# Novel X-Linked Inhibitor of Apoptosis Mutation in Very Early-Onset Inflammatory Bowel Disease Child Successfully Treated with HLA-Haploidentical Hemapoietic Stem Cells Transplant after Removal of αβ^+^ T and B Cells

**DOI:** 10.3389/fimmu.2017.01893

**Published:** 2017-12-22

**Authors:** Cristina Cifaldi, Maria Chiriaco, Gigliola Di Matteo, Silvia Di Cesare, Scarselli Alessia, Paola De Angelis, Francesca Rea, Giulia Angelino, Maria Pastore, Valentina Ferradini, Daria Pagliara, Caterina Cancrini, Paolo Rossi, Alice Bertaina, Andrea Finocchi

**Affiliations:** ^1^University Department of Pediatrics, Unit of Immunology and Infectious Diseases, Bambino Gesù Children’s Hospital, Rome, Italy; ^2^Department of Systems Medicine, University of Rome Tor Vergata, Rome, Italy; ^3^Digestive Surgery and Endoscopy Unit, Bambino Gesù Children’s Hospital, IRCCS, Rome, Italy; ^4^Division of Pediatrics, Casa Sollievo della Sofferenza Hospital, IRCCS, San Giovanni Rotondo, Foggia, Italy; ^5^Department of Biomedicine and Prevention, University Tor Vergata Rome, Rome, Italy; ^6^Department of Pediatric Hematology and Oncology, Bambino Gesù Children’s Hospital, Rome, Italy

**Keywords:** novel X-linked inhibitor of apoptosis mutation, very early-onset inflammatory bowel disease, immunodeficiency, hemapoietic stem cells transplant, immune and gastrointestinal recovery

## Abstract

Monogenic defects in genes related to primary immunodeficiencies can be responsible for inflammatory bowel disease (IBD). Mutations in the X-linked inhibitor of apoptosis (*XIAP*) gene have been described in several patients suffering from IBD and, in particular, with very early-onset inflammatory bowel disease (VEOIBD) features. We report a VEOIBD child with a novel *XIAP* gene mutation characterized by a complicated disease course, which is unresponsive to several medical treatment options. A next-generation sequencing was performed and revealed a *de novo* hemizygous mutation in *XIAP* gene: c.565T>C p.L189P. After mutation discovery, we investigated the XIAP protein expression and nucleotide-binding oligomerization domain protein 2 (NOD2) signaling by western blotting. Flow-cytometry was used to analyze intracellular protein expression in different cell subsets and T cell apoptosis. We observed reduced protein expression in lymphocytes, granulocytes, monocytes, an Epstein–Barr virus-immortalized B cell line as well as increased apoptosis, and impairment in NOD2 signaling. The child was successfully treated with HLA-haploidentical hemapoietic stem cells transplant, acquired from his mother, after *ex vivo* elimination of α/β T cells and CD19 B cells. One year after the transplant, we repeated the analysis to appreciate the changes in his impairments. The recovery of XIAP protein expression, function, and normalization of apoptosis were observed. Our report emphasizes the important role of genetic analysis in the diagnosis of VEOIBD, illustrates the complete immunological and gastrointestinal recovery after transplant, and shows one of the few successful transplant cases of XIAP patients.

## Highlights

- Very early-onset IBD patient
- Patient unresponsive to several medical treatment
- Next-generation sequencing reveals a new XIAP mutation
- The protein expression is reduced, but detectable
- Functional assays shows impaired NOD2 pathway
- XIAP protein expression and NOD2 pathway were compromised
- The HSCT led to a total immune and gastrointestinal healing

## Introduction

Inflammatory bowel disease (IBD) is a heterogeneous group of chronic inflammatory disorders affecting the gastrointestinal (GI) tract, including Crohn’s disease (CD) and ulcerative colitis. Both A chronic and relapsing inflammatory response against the intestinal microbiota is characteristic of both disorders.

The pathogenesis of IBD is complex and often associated with genetic predisposition, environmental factors, and/or epithelial barrier dysfunction, which cause persistent activation of the intestinal immune response (1–3). Primary immunodeficiencies (PIDs) may be the cause of the disease in a relevant portion of cases.

X-linked inhibitor of apoptosis (XIAP) inactivating mutations have been identified as cause for a rare X-linked immune disorder 2 (XLP2), which similarly to X-linked immune disorder 1 (XLP1), is caused by mutation in SH2 domain protein 1A gene. Classical XLP (XLP1) is characterized by susceptibility to Epstein–Barr virus (EBV) infection, frequently leading to hemophagocytic lymphohistiocytosis (HLH), with major clinical phenotypes including fulminant infectious mononucleosis, lymphoproliferative disorders, and dysgammaglobulinemia. By contrast, XLP2 is characterized by susceptibility to EBV and cytomegalovirus (CMV) infection, recurrent splenomegaly, chronic intestinal inflammation, risk of HLH, variable hypogammaglobulinemia, and auto-inflammatory manifestations. No case of lymphoma has been reported (4–15). XIAP mediates the signaling of nucleotide-binding oligomerization domain nucleotide-binding oligomerization domain protein 1 (NOD1)/nucleotide-binding oligomerization domain protein 2 (NOD2) in response to bacterial pathogens and regulates tumor necrosis factor (TNF)-mediated survival, inflammatory, and death-signaling pathways. XIAP is also a direct inhibitor of initiator and effector caspases.

In the last years, the proteins members of the inhibitor of apoptosis (IAP) family (cIAP1, cIAP2, and XIAP) with E3 ubiquitin ligase activity have been shown to mediate NOD2 signaling. Their mutations are commonly associated with IBD. XIAP is characterized by three baculoviral IAP repeat (BIR) homology domains, which are necessary to bind the TAK-binding protein 1 (TAB1), which, in turn, activates the nuclear factor light chain enhancer of activated B cells [nuclear factor kappa B (NF-κB)] pathway, suppressing specific cell death-inducing caspases, such as caspase 3, 9, and 7, *via* its BIR2 and BIR3 domains (3, 16).

In this case report, we describe a patient with very early-onset inflammatory bowel disease (VEOIBD) who presented with EBV viremia and a complicated disease course, which was unresponsive to several lines of medical treatment options. The patient showed a novel and *de novo* hemizygous mutation in XIAP gene. Functional studies revealed a reduction of protein expression in an EBV-B cell line and in the lymphocytes, granulocytes, and monocytes subsets. The suspicion of a defective NOD2 pathway was demonstrated by the lack of IκBα degradation and an increased activation-induced cell death in peripheral blood mononuclear cell (PBMC). The patient received haplo-hemapoietic stem cells transplant (HSCT) after negative depletion of Tαβ^+^/CD19^+^ lymphocytes with a complete immune-GI recovery.

## Case Report

We describe a 7-year-old first-born male patient from non-consanguineous parents. The child was in good clinical health until 5 years of age, when, he presented with mucohemorrhagic diarrhea, fever, and abdominal pain, for which he was hospitalized with a suspicion of VEOIBD. Blood tests revealed anemia and elevation of inflammatory markers. The fecal calprotectin level was significantly increased.

While the upper gastrointestinal endoscopy was normal, the colonoscopy revealed diffuse colitis, without involvement of the small intestine. Standard antibiotic (ciprofloxacin and metronidazole) and steroid treatment (prednisone 1 mg/kg) were administered with transitory clinical benefit, followed by relapse of mucohemorrhagic diarrhea during steroid taper.

**Figure 1:**
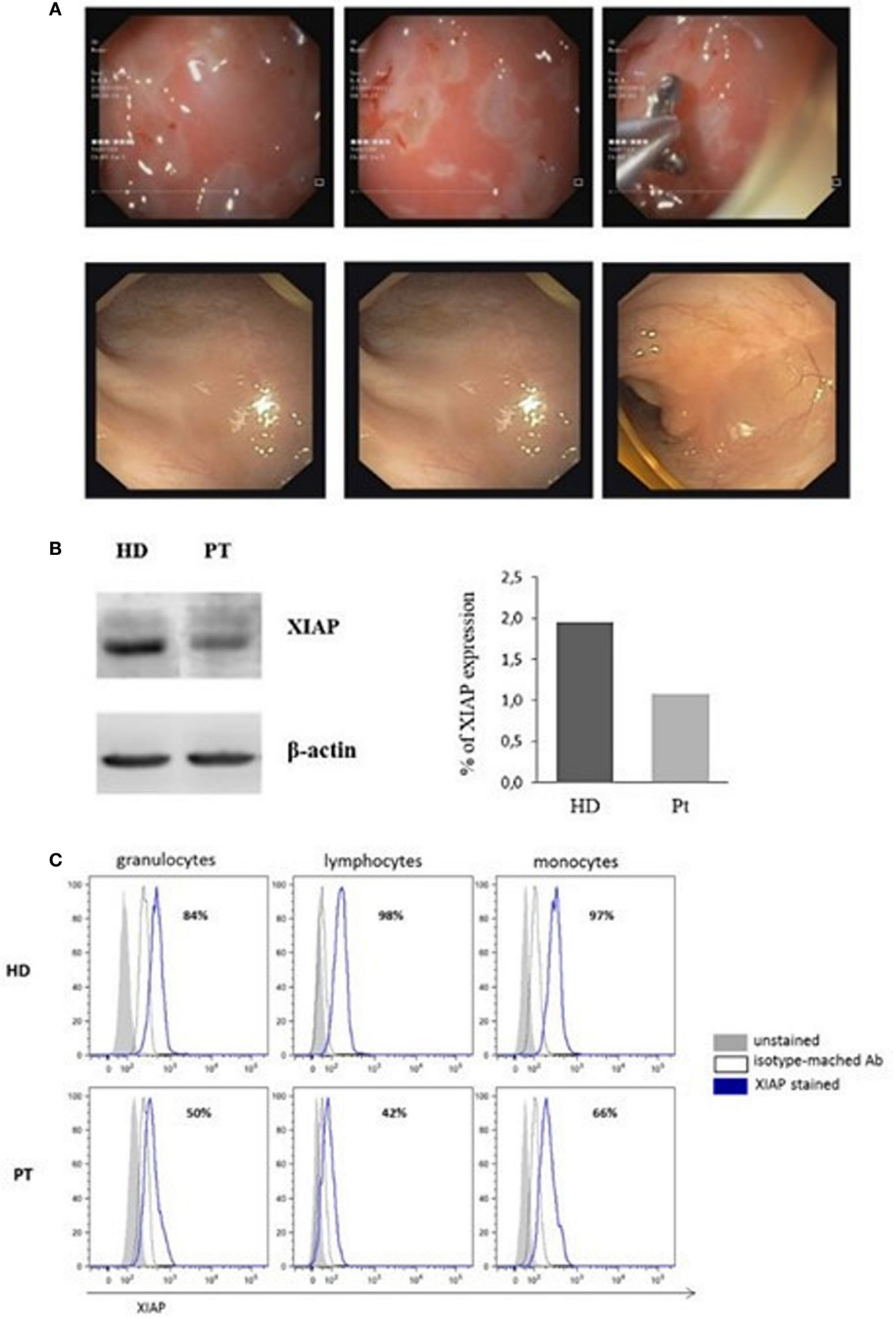
Image of colonoscopy investigation and X-linked inhibitor of apoptosis (XIAP) expression. **(A)** Colonoscopy features of the patient show the intensely erythematous mucosa, with prepyloric erosions, ulcerations covered with fibrin and intestinal chronic inflammatory process, with colonic histiocytic clusters (Top panels). Colonoscopy investigation 6 months after hemapoietic stem cells transplant revealed normal gastrointestinal and colonic mucosa with regular vascular patter (Bottom panels). **(B)** XIAP immunoblot in patient B-Epstein–Barr virus-cell line compared with healthy donor. Lysate were immunoblotted with anti-XIAP antibody and anti-β-actin as a loading control. Data are presented as percentage of protein expression. **(C)** Flow-cytometric histograms of XIAP expression. Assay was analyzed by means of intracellular staining lymphocytes, granulocytes, and monocytes in a healthy donor and XIAP patient.

Full endoscopic evaluation performed in our department revealed: intensely erythematous gastric mucosa with prepyloric erosions. The colonoscopy of the mucosa of the transverse colon, the left sigmoid colon, and the rectum appeared erythematous with ulcerations covered with fibrin. The biopsy indicated esophagitis, gastritis, and intestinal chronic inflammatory process with colonic histiocytic clusters (Figure 1A, top panels).

The child underwent an additional course of prednisone (2 mg/kg) and began azathioprine (up to a dosage of 2.5 mg/kg), for his apparent steroid dependence, with partial clinical benefit. At the same time, during episodes of fever and diarrhea, he received several treatments with metronidazole and/or ciprofloxacin, with a transient positive response, although all fecal cultures were negative.

Given the early onset of intestinal symptoms and the clinical severity, we performed a complete immunological investigation to exclude possible underlying primary immune defects. He had a mild decrease of IgG serum levels with poor antibody responses to tetanus toxoid, *Haemophilus influenzae* type b (Hib) and hepatitis B. Therefore, he was started on intravenous immunoglobulin treatment at a dose of 400 mg/kg every 3 weeks.

Of note, the patient showed persistent EBV viremia ranging from 1,218 to 32,466 copies/ml and absent seroconversion (no anti-EBNA production). A repeated colonoscopy confirmed severe pancolitis (macroscopic and microscopic findings) and PCR of DNA extracts from biopsies yielded a positive result for EBV DNA. Accordingly, the patient was administered rituximab; however, he did not tolerate therapy.

His disease course remained severe, complicated by infections, including adenovirus enteritis and pneumonia (with pleural effusion). The patient exhibited refusal of food and chronic malnutrition, despite support with polymeric formula and sometimes parenteral nutrition. Humira (80–40–20 mg) was administered twice per month for 4 months with no evidence of clinical remission.

In the meantime, next-generation sequencing (NGS) revealed a *de novo* and novel mutation in the *XIAP* gene: c.565T>C p.L189P, which, SIFT and Polyphen 2, predicted to be damaging and probably damaging, respectively. Sanger sequencing confirmed the mutation in the patient and his parents (on DNA extracted both from peripheral blood and from mouth rinsing to assess the germline mutations); however, the substitution was found only in the patient.

Since the L189P is located in the BIR2 domain of XIAP, to establish if BIR2 mutation impairs protein expression, PBMCs isolated from the patient were cultured and transformed into an EBV-immortalized B-cell line, then immunoblotted for XIAP. Reduced protein expression was observed in cells from the patient compared with a healthy control (Figure 1B).

**Figure 2:**
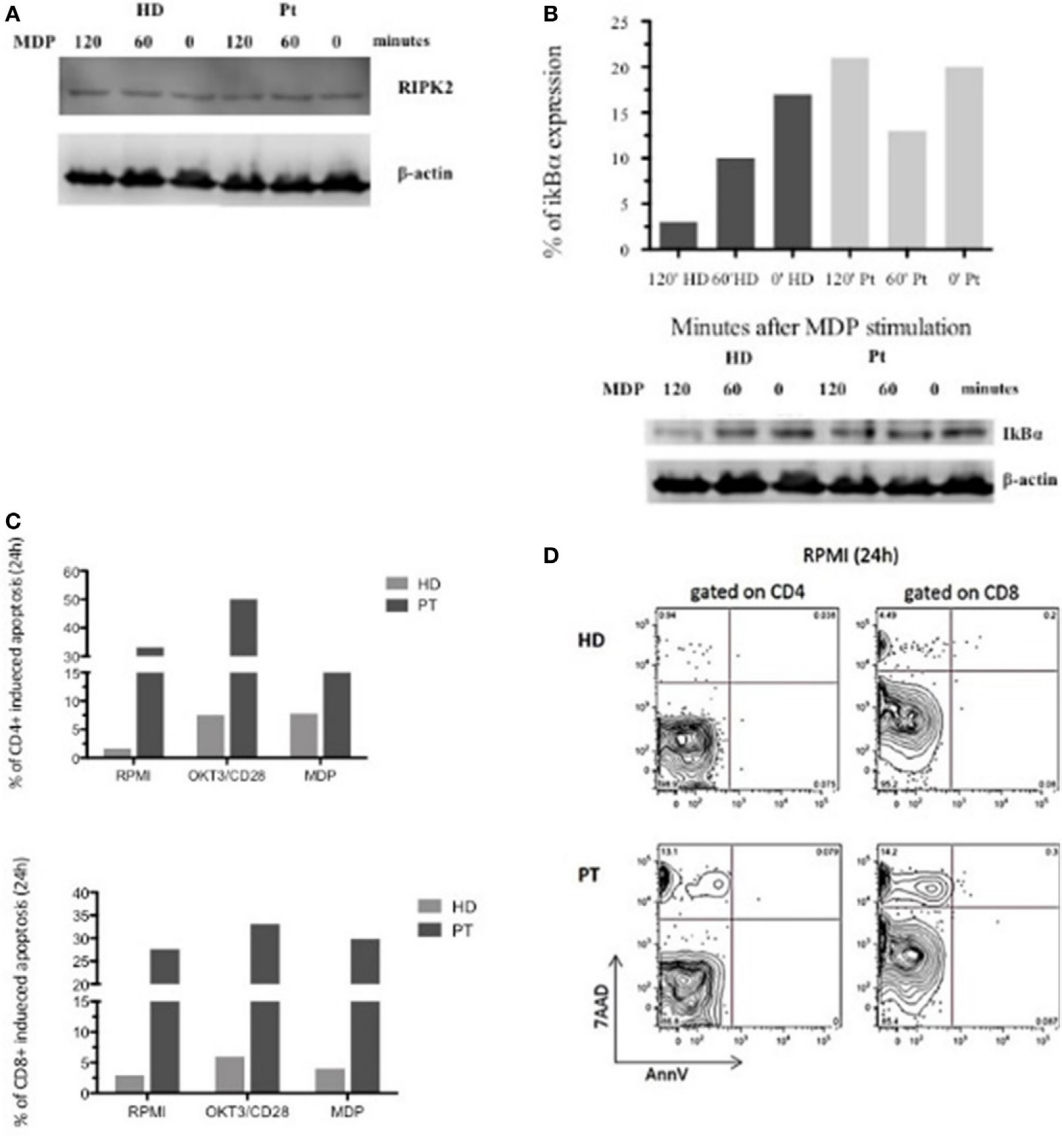
Defective nuclear factor kappa B activation in X-linked inhibitor of apoptosis-deficient patient in response to nucleotide-binding oligomerization domain protein 2 stimulation and flow-cytometric cell-death assay. **(A,B)** RIPK2 expression and impaired IκBα degradation in patient and healthy donor Epstein–Barr virus-B-cell line. Lysate were analyzed for anti-IκBα, anti-RIPK2, and loading control anti-β-actin. **(C)** *In vitro* apoptosis assay in CD4^+^ and CD8^+^ T cells from patient’s peripheral blood mononuclear cell stimulated with OKT3/CD28 or muramyl dipeptide (MDP) for 24 h. Histograms represent the mean of three independent experiments. **(D)** T cell gated on CD4^+^ and CD8^+^ unstimulated stimulated for 24 h and analyzed by 7AAD expression and Annexin V.

Afterward, we investigated the XIAP expression in different cell subsets. In accordance with our result, intracellular XIAP expression was markedly reduced in lymphocytes, granulocytes, and monocytes (Figure 1C). Typically, the NOD2 activation and the XIAP/RIPK2 recruitment lead to the NF-κB translocation to the nucleus after IκBα degradation. To determine the effect of this mutation on NOD2 pathway, we investigated the XIAP direct-binding protein, RIPK2, which did not reveal alterations (Figure 2A). In addition, we studied the IκBα degradation in patient’s EBV-immortalized B-cell line stimulated with muramyl dipeptide (MDP), a specific NOD2-activating ligand. Consistent with the data, after stimulation, IκBα degradation was absent, suggesting defective NF-κB activation (Figure 2B).

Since XIAP is a potent and direct caspase inhibitor and an antiapoptotic protein, we decided to investigate whether the patient’s mutation affects this function. We performed an *in vitro* apoptosis assay in CD4^+^ and CD8^+^ T cells from the patient’s PBMC stimulated with OKT3/CD28 or MDP for 24 h. MDP-stimulated CD4^+^ and CD8^+^ T cells had the same features of OKT3/CD28 stimulated T cells, with further propensity to apoptosis compared with a healthy control. Interestingly, analysis of the patient’s cells revealed an increased level of basal apoptosis without stimulation (Figures 2C,D). Our patient exhibited increased activation-induced cell death, which may reflect the alteration of NOD2 signaling and the inability of the XIAP protein to inhibit the caspases.

Considering the unresponsiveness to several different lines of therapy, and since the patient did not have any suitable related or fully allelic-matched unrelated donor, we decided to perform HLA-haploidentical HSCT (haplo-HSCT) after negative depletion of Tαβ^+^/CD19^+^ lymphocytes, using the mother as donor. This type of allograft permits CD34^+^ cells, committed hematopoietic progenitor cells, fully functioning donor NK and γδ^+^ T cells to remain in the graft. Recent studies have shown that Tαβ^+^/CD19^+^ cell depleted haplo-HSCT is a suitable option for the definitive treatment of a wide spectrum of malignant and non-malignant disorders, in the absence of an HLA-identical donor. This technique allows a rapid engraftment, low graft failure rate, and low incidence of significant aGvHD (17, 18). The child was included in clinical trial protocol (NCT02065869), which was approved by the ethical committee of Bambino Gesù Children’s Hospital. This treatment regimen consisted of myeloablative [myeloablative conditioning (MAC)], but reduced-toxicity conditioning, including the combination of thiotepa (8 mg/kg), treosulfan (42 g/m^2^ over 3 days), fludarabine (160 mg/m^2^ over 4 days), and anti-T-lymphocyte globulin (ATLG, Grafalon, Neovii) to prevent graft-versus-host disease (GvHD) and graft failure. One single dose of rituximab (200 mg/mq) on day −1 was employed for preventing EBV-related posttransplant lymphoproliferative disorder. The patients did not receive any post-HSCT pharmacological GvHD prophylaxis. The median time to reach neutrophil and platelet engraftment was 13 and 11 days, respectively, and he achieved a full donor chimerism. No toxicities or serious adverse events occurred.

Endoscopic evaluation 3 and 6 months after HSCT showed normal upper GI mucosa, normal colonic mucosa with regular vascular patter, a cecal pseudo-polyp (histology: no signs of GvHD, mild esophagitis, and signs of previous colonic inflammation with regeneration) (Figure 1A, bottom panels). At present time, the child is 1-year post-HSCT and is in very good general condition with full donor chimerism.

**Figure 3:**
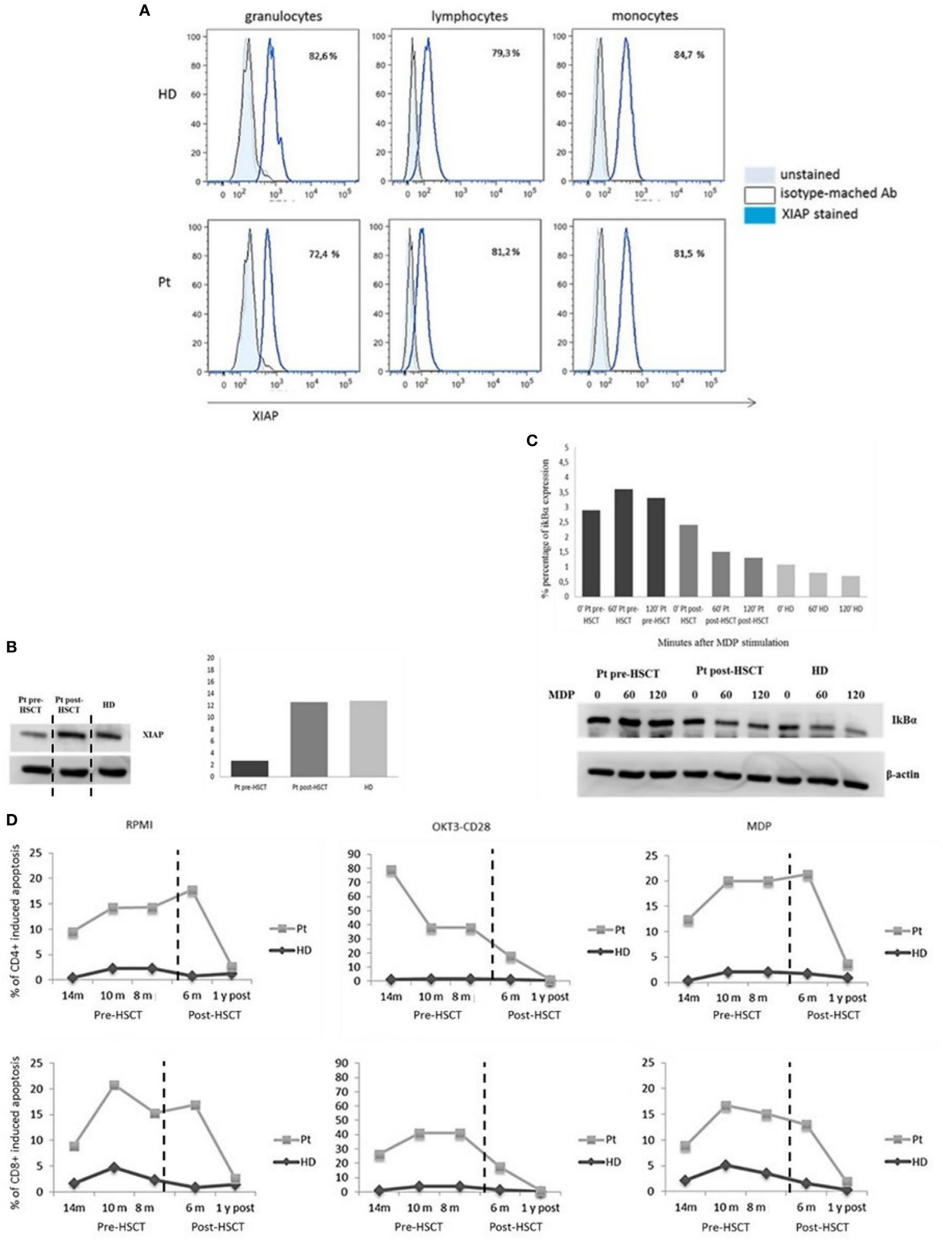
X-linked inhibitor of apoptosis (XIAP) expression. **(A)** Flow-cytometric XIAP expression 1-year post-hemapoietic stem cells transplant (HSCT). **(B)** Reestablished XIAP immunoblot in patient B-Epstein–Barr virus-cell line compared with healthy donor. The XIAP expression has been derived from the same IκBα immunoblot **(C)** incubated with anti-XIAP. The only time point 0 is showed. Data are presented as percentage of protein expression. Dashed vertical lines separate the time point 0 showed in panel **(B)** for XIAP immunoblot. **(C)** Restored IκBα degradation in response to nucleotide-binding oligomerization domain protein 2 stimulation. **(D)** Time course of pre/post-HSCT cell-death assay. T cell gated on CD4^+^ and CD8^+^ unstimulated, OKT3/CD28, or muramyl dipeptide (MDP) stimulated for 24 h and analyzed by 7AAD expression.

One year after transplant, we analyzed again the expression of XIAP by intracellular staining and western blotting (Figures 3A,B) again. The protein expression was restored in all leukocyte subsets and the EBV-B-cell line. Moreover, IκBα degradation was detected, suggesting a reinstated NF-κB activation (Figure 3C). See Figure 3 legend. Finally, 6 months and 1 year following HSCT, we performed the apoptosis assay to evaluate the antiapoptotic capacity, which was comparable to healthy donor (Figure 3D).

## Discussion

Inflammatory bowel disease has been identified as a possible sign of several recently discovered PIDs; it may be a clinical feature of a multisystemic disease or a consequence of epithelial barrier defects, phagocytic defects, B and T cell abnormalities (severe combined immunodeficiency, common variable immunodeficiency, Wiskott–Aldrich syndrome), autoimmunity (XLP2), and IL10R defects (19–21).

X-linked inhibitor of apoptosis has an important role downstream of the NOD1 and NOD2 immune receptors (22–24) and as previously reported, mutations in NOD2 are a predisposition to the development of CD (1, 25). These receptors are expressed primarily in monocytes and induce activation of mitogen activation protein kinases and NF-κB, leading to production of pro-inflammatory cytokines, such as TNF, interleukin (IL)-1β, IL-6, and IL-8, and various antimicrobial peptides (26, 27). Monocytes from patients with XIAP deficiency have an impaired ability to secrete cytokines in response to stimulation by NOD2 ligands.

Upon binding of MDP to the NOD2-receptor, the receptor-interacting protein 2 (RIP2) is recruited with IAPs proteins. This complex ubiquitinates RIP2, which acts as a scaffold for TAK1 and TAK-binding protein 2/TAK-binding protein 3 together with IκB kinase and linear ubiquitin chain assembly complex. As a result, IκBα is phosphorylated and targeted for degradation, releasing NF-κB (3, 28–30).

Interestingly, a study by Damgaard et al., which analyzed the function of XIAP mutations from XLP2 individuals, revealed that they have defective NOD2 signaling. This may explain the association of IBD with XLP2 (3, 6).

X-linked inhibitor of apoptosis protein, as an antiapoptotic molecule, consists of 497 amino acids containing three baculovirus IAP repeat domains (BIR1, BIR2, and BIR3) and one RING and UBA domain, respectively. These BIR domains allow the binding of XIAP to caspases 3, 7, and 9 inhibiting the proteolytic activity of caspases 3, 7, and 9 (31). The increased apoptosis basally and after stimulation, seen in our patient, likely reflects the impaired function of the BIR2 domain to bind and inhibit caspases. Moreover, we could hypothesize that the L189P mutation prevents the binding of XIAP with RIP2 protein altering the downstream signal transduction and the activation of NF-κB, as seen in the patient.

Most of XIAP mutations described in XLP2 (such as nonsense and frameshift mutations or deletions) fall throughout the protein length leading to severe aberrations in the encoded protein and loss of expression. On the contrary, missense mutations cluster in two hotspots, the BIR2 and RING domains exactly like how L189P mutation (6).

Recent observations reveal that IBD due to XIAP defect is most commonly a pediatric disease presenting in the first years of life. Pediatric patients often present with more severe symptoms and a more aggressive disease course than is observed in adult patients. This disease negatively affects the quality of life of young patients in which the impact of the disease on growth and nutrition necessitates careful management to ensure physical and psychosocial functioning (12). While there are no significant immunologic abnormalities, the clinical manifestations including HLH, recurrent splenomegaly, and IBD are severe. These patients are susceptible to EBV and CMV infection, which could be explained by defects in adaptive immunity affecting T-lymphocyte responses—a critical immune response to viral infections (11). As described earlier, our patient showed mild humoral abnormality and persistent EBV viremia without evidence of HLH. Despite the absence of major immunological features, several defects associated with XIAP deficiency have been demonstrated *in vitro* (11). Our data demonstrate the compromised XIAP protein expression and function in the NOD2 signaling. This process may cause an altered production of pro-inflammatory cytokines as well as the autophagic elimination of intracellular bacteria. IBD is generally severe and drug-resistant with a fatal outcome in the lesser part of the cases, with the only possible curative treatment being HSCT. Nevertheless, HSCT in XIAP patients has been associated initially with bad prognosis compared with slam-associated protein deficiency and there are a limited number of studies concerning the outcomes of HSCT in patients with XIAP deficiency (32–39). While HSCT may be supported for patients with inherited immunodeficiencies that cause HLH (due to the life-threatening nature of HLH), the complicated course of some patients warrants precaution with this procedure (32, 33). In terms of allo-HSCT for XIAP deficiency, Marsh et al. described the international experience in 19 patients showing an extremely high transplant-related mortality in those prepared with an MAC (7/8 patients died), in comparison with those receiving a reduced-intensity conditioning (RIC) (5/11 patients died) (32). Thus, in this setting, the use of MAC has been associated with significantly higher mortality. It is possible that the loss of XIAP and its antiapoptotic functions contributes to the high incidence of treatment-related toxicities observed with MAC regimens (16). Indeed, patients transplanted with MAC regimens have a survival of approximately 60% and a risk of death for toxicities and complications including pulmonary hemorrhage, pulmonary hypertension, GvHD, sepsis, and multiorgan failure. However, one case of a patient who received MAC HSCT with a good follow-up and free of disease is reported (35). On the contrary, patients who received RIC had a survival rate of approximately 80% (35–37). Overall, the choice of HSCT for XIAP deficiency reveals poor outcomes and there is a propensity toward an RIC regime. The absence of HLH, in our patient, was likely responsible for the success of the transplant. The presence of HLH is an important factor in the choice of conditioning. The goal of transplantation in patients with XIAP deficiency is to establish sufficient donor cell engraftment to prevent HLH recurrence; however, there is no consensus regarding the lowest level of donor chimerism required to prevent disease relapse following HSCT. It is prudent to closely monitor chimerism in these patients’ posttransplant period, due to an increased risk of mixed chimerism. This is consistent with earlier findings suggesting that whole blood donor chimerism greater than 20% appears to be sufficient to protect against HLH recurrence (35, 38).

## Concluding Remarks

We herein described a child with VEOIBD with a novel XIAP mutation, who received a TCRαβ^+^ T cell- and CD19^+^ B cell-depleted haploidentical HSCT from his maternal donor. During the short follow-up period, a complete immune and GI recovery was observed, although a long-term follow-up is required to confirm the reconstitution. Genetic testing, in particular for atypical presentations, and early diagnosis are fundamental to prevent the worsening of XIAP disease. Therefore, XIAP should be suspected for those forms of early onset of IBD in which a prompt diagnosis may allow an appropriate treatment (i.e., HSCT) for the purpose of optimal management of the patient.

## Ethics Statement

All procedures performed in the study were in accordance with the ethical standards of the institutional research committee and with the 1964 Helsinki declaration and its later amendments or comparable ethical standards. Informed consent was obtained from the patient and was approved by the Institutional Ethical Committee of Ospedale Pediatrico Bambino Gesù and signed by his parents.

## Author Contributions

CC designed and performed experiments, analyzed the data, and wrote the paper; MC, GM, and SC performed laboratory analysis and contributed to data collection, interpretation, and revision of the manuscript; VF, GM, and CC performed the NGS and the bioinformatic analyses; SA, PA, FR, GA, MP, DP, CC, PR, AB, and AF have been important part in complicated diagnostic and therapeutic course and give valuable interpretation of data. AF supervised the project. All the coauthors revised paper critically and gave final approval of this version for publishing.

## Conflict of Interest Statement

All authors declare that the research was conducted in the absence of any commercial or financial relationships that could be construed as a potential conflict of interest.

## Abbreviations

PID, primary immunodeficiency; IBD, inflammatory bowel disease; CD, Crohn’s disease; UC, ulcerative colitis; VEOIBD, very early-onset inflammatory bowel disease; XLP1, X-linked immune disorder 1; XLP2, X-linked immune disorder 2; XIAP, X-linked inhibitor of apoptosis; NOD1, nucleotide-binding oligomerization domain protein 1; NOD2, nucleotide-binding oligomerization domain protein 2; SH2D1A, SH2 domain protein 1A; CMV, cytomegalovirus; EBV, Epstein–Barr virus; Hib, *Haemophilus influenzae* type b; IVIG, intravenous immunoglobulin treatment; ATLG, anti-T-lymphocyte globulin; GvHD, graft-versus-host disease; GI, gastrointestinal; UGI, upper gastrointestinal endoscopy; HLH, hemophagocytic lymphohistiocytosis; TNF, tumor necrosis factor; HSCT, hemapoietic stem cells transplant; NGS, next-generation sequencing; PBMC, peripheral blood mononuclear cell; MDP, muramyl dipeptide; IL-1β, interleukin 1 beta; IL-6, interleukin 6; IL-8, interleukin 8; IAP, inhibitor of apoptosis proteins; BIR, baculoviral IAP repeat homology domains; TAB1, TAK-binding protein 1; TAB2, TAK-binding protein 2; TAB3, TAK-binding protein 3; IKK, IκB kinase; MAP, mitogen activation protein; IκBα, inhibitor of kappa light chain gene enhancer in B cells, alpha; NF-κB, nuclear factor kappa B; LUBAC, linear ubiquitin chain assembly complex; SAP, slam-associated protein; TRM, transplant-related mortality; RIC, reduced-intensity conditioning; MAC, myeloablative conditioning.
